# Supplementary material for: Exposure to Bacillus cereus in Water Buffalo Mozzarella Cheese
Source: Foods. 2020 Dec 19;9(12):1899. doi: 10.3390/foods9121899 (PMC7766095; doi:10.3390/foods9121899)
Supplement: Supplementary file 1 [file foods-09-01899-s001.pdf]

Table S1. *B. cereus*'s maximum growth rate (log.conc/h), double time (hours), maximum population density (MPD; log CFU/g) and lag time (hours) estimated through ComBase predictor software (www.combase.cc) from an initial contamination level of 2 Log CFU/g and 3 Log CFU/g in water buffalo mozzarella cheese (with a pH 5.7 and an a_w 0.997) at three temperature (15, 18 and 22°C).

Init. Level	Phys.state	pH	AW	Temp (°C)	Max.rate (log.conc/h)	Dbl. time (Hours)	MPD (log CFU/g)	Lag time (Hours)
2<br>3	2.7×10^{-4}	5.7	0.997	15	0.105	2.876	7.61	33.92
				18	0.16	1.878	7.61	22.26
				22	0.268	1.125	7.61	13.29
